# Metabonomic and transcriptomic profiling reveals amino acid metabolism affects the quality of premium japonica rice varieties in Northeast China

**DOI:** 10.1016/j.fochms.2024.100230

**Published:** 2024-11-20

**Authors:** Jing Wang, Haitao Guan, Xiaolei Zhang, Changjun Dai, Cuiling Wang, Guofeng Chen, Kun Li, Zhenhua Xu, Ruiying Zhang, Baohai Liu, Hongtao Wen

**Affiliations:** a Quality and Safety Institute of Agricultural Products, Heilongjiang Academy of Agricultural Sciences/Key Laboratory of Quality and Safety of Cereals and Their Products, State Administration for Market Regulation, Harbin 150086, China; b Biotechnology Research Institute, Heilongjiang Academy of Agricultural Sciences, Harbin 150086, China

**Keywords:** *Oryza sativa* L., Metabolomics, Transcriptomics profiles, Rice quality

## Abstract

Rice consumption and demand for premium rice are increasing worldwide. However, the characterizations and how to identify the premium rice are still unclear. Small molecular metabolites have a great advantage in distinguishing subtle differences among similar agricultural products. So, we hypothesized that the metabolites would be the key to identifying the tiny differences in premium rice among similar varieties. In this study, we performed metabolomic and transcriptomic profiles to comprehensively elucidate key metabolites, genes, and formation mechanisms of premium rice. As a result, eight compounds belong to four categories, and 49 different expressional genes were identified in premium rice varieties after comparing with the second-best varieties. Moreover, the integrated analysis confirmed that the amino acid pathway, including 42 expression genes and 11 metabolites, was critical for the premium rice formation. Six genes and two metabolites had significant regulatory effects on the pathways. Furthermore, amino acid quantification confirmed the content of 12 kinds of hydrolytic amino acids, such as aspartic acid and arginine were different between premium and other varieties. These amino acids may serve as potential biomarkers for differentiating premium rice in Northeast China. Our results strongly support the possibility of differentiating premium rice and would provide essential data for premium rice identification and metabolomics-assisted breeding.

## Highlights

• Multi-omics analysis revealed the distinctions between premium and second-best rice.
• A total of 8 metabolites and 49 genes were identified between these two rice types.
• These metabolites include amino acid, diterpenoid, phenolic acid, and saccharide.
• The genes are linked to organonitrogen compound metabolic and biosynthetic processes.
• Amino acid pathways are crucial in premium rice formation and distinction.

## 1 Introduction

Rice (*Oryza sativa* L.) is the primary food source for nearly half the global population. Due to social economy advancements and the expanding global reach of the international rice market, food security is required to develop a high-quality rice industry (Custodio et al., 2019; Liu, Jiang, et al., 2022; Xiong et al., 2022). High-quality products, characterized by their palatability and aromatic properties, typically command a price of two or three times higher than ordinary ones. So, food fraud like a combination of other rice varieties with high-quality rice does not significantly influence the flavor, color, or form, because the structures and nutritional ingredients of different kinds of rice are quite similar (Liu, Li, et al., 2020). Every year, over 4 million tons of Japonica rice are transported out of Northeast China as a product and most of them are high-quality rice products (Charoenthaikij et al., 2021; Zhao et al., 2021). However, the limited standards are insufficient for identifying high-quality rice varieties, let alone the differentiation of similar varieties. Therefore, it is urgent to develop an effective method to ascertain whether there are discernible differences between premium and second-best varieties and whether these distinctions are prevalent in the former.

Rice quality evaluations, including morphological characteristics, physicochemical analysis, and sensory properties of flavor and palatability, mainly depend on quantifying a limited number of compounds or subjective and costly human measurements (Zheng et al., 2022; Zhu et al., 2023). Indeed, descriptive sensory analysis is a simple method for differentiating similar varieties (Champagne et al., 2010). Since different sensory attributes depend on subjective evaluation, inaccuracies cannot be eliminated (Custodio et al., 2019). Therefore, the nuances of premium japonica rice in Northeast China must be discerned through more reliable means for assessing rice quality characters. Metabolites are by-products of gene and protein regulation, their composition and abundance influenced the phenotype of rice (Zeng et al., 2021). Non-target metabolomics, a comprehensive and meticulous investigation of metabolites within biological systems, can qualitatively and quantitatively elucidate the variation of metabolites in highly similar varieties (Zhao, Duan, et al., 2022). The application of this method to study metabolite differences has proven fruitful results (Daygon et al., 2017). For example, it has been used to discriminate Wuchang Dahuaxiang 2 rice (Zhao et al., 2023; Zhao, Liu, et al., 2022). Metabolites provide an alternative point of view for comparing the subtle differences between premium rice varieties and may serve as the foundation and immediate reflection of an organism's phenotype.

Recently, metabolomics combined with transcriptomics and multivariate statistics has provided insights into the specific types and amounts of metabolites present during rice growth (Liu, Liu, et al., 2020). Transcriptome sequencing represents a valuable tool for obtaining gene expression data (Guo et al., 2024; Rana et al., 2022; Wang et al., 2022; Yang, Jiang, et al., 2022). Many allele genes have been identified, including *Waxy* (*Wx*), A*lkaline Denaturation* (*ALK*), *growth retardation1* (*FGR1*)*, floury endosperm7* (*FLO7*)*, Grain Length/grain width on Chromosome 7* (*GL7/GW7*)*, Grain Length, Width and Weight 7* (*GLW7*)*, grain size on chromosome 2* (*GS2*)*, GS3, ONAC129, Growth Regulating Factor8* (*OsGRF8*)*, put on weight 1* (*POW1*)*, WHITE-CORE RATE 1* (*WCR1*)*, starch synthase I* (*SSI*)*, SSII, and Oryza sativa Stress tolerance and Grain Length* (*OsSGL*) (De Vos et al., 2007; Liu, Liu, et al., 2022; Yang et al., 2013; Yang et al., 2023), and some quantitative trait loci (QTLs) (Jiang et al., 2024) have been identified as providing molecular basis for the coordinated improvement of rice quality. The integration of transcriptomic and metabolomic analysis can provide essential evidence for critical pathways to comprehensively analyze the formation mechanism of rice quality. For instance, metabonomic and transcriptomic profiling revealed the regulatory mechanism of head-milled rice rate, grain size, chalk formation, and other processes (Li, Liu, et al., 2022; Yang, Jiang, et al., 2022). Moreover, the integration analysis can simultaneously achieve qualification and quantification of a global profile of compounds, such as nutritional composition (Ramalingam et al., 2021; Xiao et al., 2021) and potential biomarkers (Galindo-Prieto et al., 2015; Setyaningsih et al., 2019)). Consequently, it effectively shows the minute clues within the rice and the underlying metabolic mechanism.

In this study, we employed non-targeted metabolomic and transcriptomic technology to investigate the relationships between types of metabolites and gene expression in premium and second-best japonica rice varieties grown in Northeast China. This study further investigated the crucial metabolites and genes providing primary data for high-quality rice identification and metabolism-assisted breeding.

## 2 Materials and methods

### 2.1 Materials and reagents

Two high-quality rice varieties, WuYouDao 4 (WYD4) and JiYuanXing1 (JYX1), two second-best varieties, Suijing 18 (SJ18) and Longjing31 (LJ31), were selected in this study (https://www.ricedata.cn/). All of them were planted in the largest area in their accumulated temperature of Northeast China. Following the local requirements for high-yield paddy rice cultivation, measurements were implemented for water management, disease, pest, and weed prevention and control during the planting process. Paddy rice was harvested after maturity and stored at the Heilongjiang Academy of Agricultural Sciences at a temperature below 15 °C. The rice quality was evaluated without delay to stabilize the physical and chemical properties of the rice samples. The weight of the rice was assessed using the wet weight method. Following the removal of the milled rice from storage, it was maintained in an environmentally controlled room at a constant temperature and humidity (temperature = 28 °C humidity <10 %) for one week to ensure uniform moisture content (approximately 14 %) of all samples. Each sample contained a minimum weight of 2 kg in total (GB/T 3543.2). The authenticity and purity identification of the four rice varieties were ascertained using SSR markers. The detailed information regarding the primers and the procedure followed the standards outlined in the Chinese national standard GB/T 39917–2021. The samples for RNA sequencing were rapidly collected in liquid nitrogen and stored at −80 °C after grinding. A hulling machine (FC2K-178,046, OTAKE, Aichi, Japan) and a milling and polishing machine (Vp-32, Yamamoto, Yamagata, Japan) were used for hulling and milling rice for subsequent experimental analysis. Different solvents and chemicals were sourced from Sigma-Aldrich (Beijing, China). Additional materials such as syringes, PVDF filters (25 mm × 0.22 μm), amber vials (2 mL), PP falcon tubes (50 mL), test tubes, glass beakers, etc. were purchased from Corning (Beijing, China).

### 2.2 Evaluation of rice quality

The grain milling quality, brown rice, milled rice, and head milled rice percentage were determined following the hulling and milling of approximately 150 g of seeds (GB/T17891–2017). The grain appearance quality, length, width, degree of endosperm chalkiness, and percentage of grain with chalkiness were determined using a rice scanning machine (SC-E, Wanshen Technology Company, Hangzhou, Zhejiang, China). The amylose contents of the milled rice were determined by the iodine colorimetric method following the pulverization of the rice samples (Grain Crusher, FS-100, Nanjing, Jiangsu, China) and their passage through a 100-mesh sieve (GB/T15683–2008) (Zhu et al., 2008). The crude protein content was determined through the Kjeldahl method (Barbano et al., 1990) using an 8400 Kjeltec analyzer (Foss Tecator, Hillerød, Denmark) following the manufacturer's instructions. The water content was determined using the direct drying method (GB5009.3–2016). The pasting properties of the rice flours and starches were evaluated using a rapid viscosity analyzer (RVA) (RVA-SUPER3, Newport Scientific, Warriewood, Australia) following the previously established method (GB/T 24852–2010) (Zhang et al., 2013). The characteristics of the samples were then measured, including peak viscosity (PV), holding viscosity (HPV), final viscosity (CPV), breakdown (BD=PV-HPV), setback (SB=CPV-PV), and paste temperature (PaT). All measurements were taken with samples from the three replications, and the mean trait value of each accession was used in the subsequent data analysis. Portions (200 g) of milled rice were washed and transferred to a rice cooker (SR-CNK05-K, Panasonic, Quzhou, Zhenjiang, China) with an appropriate water-to-rice ratio of 1:1.35 (as recommended by Champagne et al., 1999). A minimum of ten panelists who had undergone training in the principles and concepts of descriptive sensory analysis participated in the study. The procedural details for the presenting samples and standards to the panelists at each session are as previously described in the standards GB/T 15682–2008 and DB23/T 2687–2020.

### 2.3 Amino acid measurement

Amino acid quantitation was performed using the Hitachi L-8800 Amino Acid Analyzer (Tokyo, Japan). The characteristics and buffer components for this instrument followed GB 5009.124–2016. Aspartic acid, arginine, glutamate, phenylalanine, isoleucine, glycine, alanine, serine, valine, methionine, leucine, tyrosine, proline, lysine, tyrosine, and threonine samples were obtained from Shanghai ANPEL experiment technology Co. LTD (Shanghai, China).

### 2.4 Metabolite identification and data analysis

The metabolite extraction was conducted following the methodology outlined by De Vos et al. (De Vos et al., 2007). The sample extracts were analyzed using a UPLC-ESI-MS/MS system (UPLC, SCIEX, Framingham, MA, USA; MS, Applied Biosystems, Foster, CA, USA). The analytical conditions were as follows: The UPLC column was an Agilent SB-C18 (1.8 μm, 2.1 mm × 100 mm, Agilent, Shanghai, China). The solvents were water with 0.1 % formic acid (solvent A) and acetonitrile with 0.1 % formic acid (solvent B). Sample measurements were conducted using a gradient program with the following conditions: 95 % A and 5 % B condition. Over 9 min, a linear gradient to 5 % A and 95 % B was applied resulting in a composition of 5 % A and 95 % B. This composition was maintained for 1 min. Subsequently, within 1.1 min, a composition of 95 % A and 5.0 % B was established and maintained for 2.9 min. The parameters were as follows: the flow rate was 0.35 mL per minute, the column oven was maintained at 40 °C, and the injection volume was four μL. The effluent was alternatively connected to an ESI-triple quadrupole linear ion trap (QTRAP)-MS. The operating parameters were as follows: source temperature 550 °C; ion spray voltage 5500 V (positive ion mode)/−4500 V (negative ion mode); ion source gas I, gas II, and curtain gas was 0.34, 0.41, and 0.17 MPa, respectively; collision-activated dissociation was high. QQQ scans were acquired as multiple reaction monitoring (MRM) experiments with the collision gas (nitrogen) set to a medium level. Further optimization is employed to achieve the declustering potential and collision energy for individual MRM transitions. A specific set of MRM transitions was monitored for each elution period according to the metabolites present. Each variety was measured in three biological replicates.

An unsupervised principal component analysis (PCA) was conducted using the prcomp statistical function in R (www.r-project.org). Before unsupervised PCA, the data were scaled by unit variance. The hierarchical cluster analysis (HCA) results of the samples and metabolites were presented as heatmaps with dendrograms. In contrast, Pearson correlation coefficients between samples were calculated using the cor function in R and presented as heatmaps only. In the case of HCA, the normalized signal intensities of metabolites are represented as a color spectrum. The variable important in projection (VIP) values were extracted from the Orthogonal Partial Least Squares Discriminant Analysis (OPLS-DA) results, including the score, permutation, and permutation plots. The VIP values were generated using the R package MetaboAnalystR. An OPLS-DA analysis was conducted after the data made a log-transformed and mean-centering. A permutation test (200 permutations) was performed to prevent overfitting. The identified metabolites were annotated using the KEGG (Kyoto Encyclopedia of Genes and Genomes) and Compound Database (http://www.kegg.jp/kegg/compound/), mapped to the KEGG pathway database (http://www.kegg.jp/kegg/pathway.html). Subsequently, pathways with significantly regulated metabolites were subjected to metabolite set enrichment analysis (MSEA), and the hypergeometric test's *p*-values were employed to ascertain the significance of the result.

### 2.5 RNA extraction and transcriptomic analysis

Total RNA from milled rice was extracted using the Omega Plant RNA Kit (Norcross, GA, USA). The quality and integration were measured by NanoPhotometer (NP50, Implen, Munich, Germany), Qubit 2.0 (Invitrogen, Singapore, Singapore), Agilent 2100 (Agilent, Palo Alto, CA, USA), and agarose gel electrophoresis analysis (Liuyi Biotechnology, Beijing, China). The transcriptome sequence library was prepared following the instructions, using the NEBNext® UltraTM RNA LibraryPrep Kit (Ipswich, MA, USA) for Illumina (San Diego, CA, USA). CDNA libraries were sequenced by the DNA nanoball sequencing platform by Metware Biotechnology Co., Ltd. (Wuhan, China). Clean reads were obtained by removing adapter sequences and omitting reads containing more than 10 % unknown nucleotides or low-quality bases (containing more than 50 % bases with a Q-score ≤ 20). Gene expression levels were evaluated in fragments per kilobase million based on the number of fragments mapped to the reference sequence. Different expressional analyses were conducted using the DEGseq R package. Gene Ontology (GO) and KEGG pathways enrichment were analyzed through clusterProfiler R package. The four varieties of mixed milled rice were selected as three biological replicates.

### 2.6 Statistical analysis

The results of this study were initially evaluated through the analysis of variance (ANOVA) and subsequently validated through the application of Duncan's multiple-range test. The data were analyzed using GraphPad Prism version 8.0.2 (GraphPad Software, La Joola, CA, USA), and statistical significance was defined as *p* < 0.05.

## 3 Results

### 3.1 Identification and characterization of four rice varieties of Northeast China

We collected more than 700 rice samples from various rice-producing regions from 2008 to 2023 through the “Chinese Rice Festival.” The authenticity and purity of the samples were verified using the SSR marker fingerprint method (data not shown). After a comparison of the eating scores, apparent amylose content, and protein content, two premium varieties with higher eating scores, WYD4 and JYX1, were designated as the premium rice group. Similarly, SJ18 and LJ31, which exhibited an intermediate middle eating quality, were designated as second-best control. All four varieties are widely cultivated in Heilongjiang Province, China (Table S1). We identified the physicochemical properties of the four varieties. The WYD4 and SJ18 varieties exhibited long seed length, elevated aroma, higher eating scores, and apparent amylose content. WYD4 exhibited the highest scores for eating quality score, apparent amylose content, and chalkiness degree. SJ18 showed an intermediate eating score, accompanied by the lowest amylose content, the highest head rice rate, high RVA profiles, and the lowest setback. JYX1 and LJ31 were identified as short-shaped seeds with a light fragrance. Their physiochemical property values were found to be in the middle range. However, JYX1 and LJ31 were evaluated with higher and intermediate eating scores, respectively (Table 1). The results demonstrated that it is challenging to identify rice with high and medium eating quality based on physicochemical properties alone. Furthermore, these standard parameters were limited in distinguishing premium rice.

### 3.2 Metabolomics analysis of primary and secondary metabolites in premium rice

**Fig. 1:**
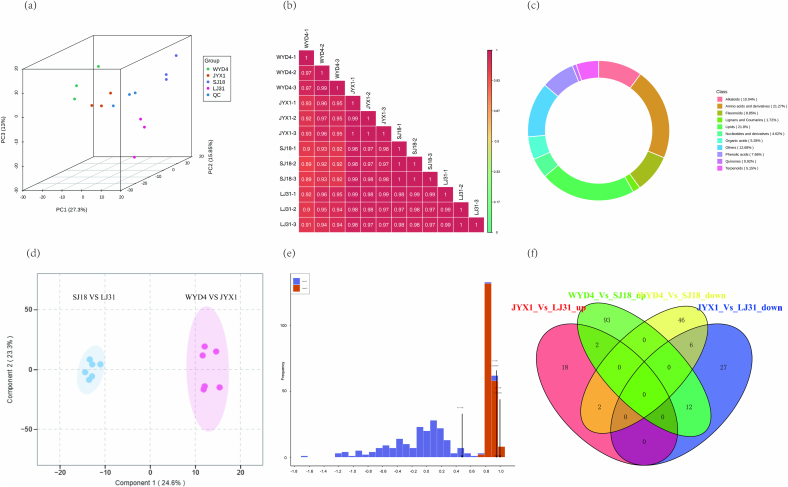
Visualization of the metabolites identified from the WYD4, JYX1, SJ18, and LJ31 varieties. (a) PCA analysis of metabolites identified from the four varieties. Each variety included three biological replicates. Equal volumes of each sample were mixed as quality control (QC). (b) The repeat correlation among the four varieties is evaluated using Pearson’ s correlation coefficient. (c) Types and proportions of the metabolites identified from four varieties. (d) OPLS-DA model of differential metabolites identified between WYD4 Vs. JYX1 and SJ18 Vs. LJ31 groups. (e) Permutation test of OPLS-DA model. (f) Venn distribution of metabolites. Different metabolites between JYX1 Vs. LJ31 and WYD4 Vs. SJ18.

According to the characterization of the four varieties, we selected WYD4-JYX1 and SJ18-LJ31 as the premium and second-best groups, respectively. Subsequently, we conducted a comparative analysis between WYD4 and SJ18, and between JYX1 and LJ31. A metabolomics analysis was conducted on the four rice varieties with the freshly milled rice. Following quality control analysis, 757 metabolites were identified and annotated (Table S2). Principle component analysis (PCA) was conducted to ascertain the contributions of PC1, PC2, and PC3, which were found to be 27.30 %, 15.85 %, and 13.00 %, respectively. The combined contribution of the two principal components was 56.10 % (Fig. 1a). The dots of the same color were from the same group, indicating clear distinctions among these four groups. According to the metabolites, JYX1 showed the closest relationship with WYD4, as did SJ18 and LJ31 (Fig. 1b). All of the compounds mentioned above were grouped into 11 categories, including lipids, amino acids and derivatives, alkaloids, flavonoids, phenolic acids, organic acids, terpenoids, nucleosides and derivatives, lignans and coumarins, quinones, and others (Fig. 1c). These results demonstrated that we obtained high-quality metabolomic data for component analysis and identification of crucial metabolites.

To identify the crucial metabolites in rice with high consumption levels, different metabolites between the premium group (WYD4-JYX1) and the second-best group (SJ18-LJ31) were selected based on log2 Fold Change ≥2 or ≤ 0.5, and VIP1 > 1. About 228 differential metabolites were identified, including 161 in the WYD4 Vs. SJ18 and 67 in the JYX1 Vs. LJ31 groups (Table S3). To evaluate these differential metabolites, OPLS-DA showed Component 1 (24.6 %) and Component 2 (23.3 %) together, which explained 57.9 % of the total variance (Fig. 1d). The permutation test demonstrated that the OPLS-DA model was both effective and stable, with Q^2^ (0.952) and R^2^Y (0.998) exceeding the 0.5 thresholds (Fig. 1e). Moreover, HCA was subsequently employed to mitigate the impact of these secondary metabolites on pattern recognition. A total of 99 metabolites were identified as being abundant in the premium group. A Venn analysis revealed that two metabolites were up-regulated and six were down-regulated in the WYD4 Vs. SJ18 and JYX1 Vs. LJ31, respectively (Fig. 1f). The compounds were classified into four categories: amino acids and derivatives, diterpenoids, phenolic acids, and saccharides. Compared to SJ18 and LJ31, the concentration of amino acids and derivatives, specifically L-arginine and Tyr-His-Leu, was lower in the premium group. Conversely, the levels of L-aspartic acid, Thr-Ile-Asp-Phe-Glu, momilactone B, 1-O-caffeoyl glycerol, and DMelezitose O-rhamnoside were higher in the group (Table 2). These results indicate that premium and second-best rice varieties contained different metabolites. Therefore, this set of metabolites may serve as potential biomarkers for identifying premium rice.

### 3.3 Transcriptomic analysis of the different expressional genes in premium rice

**Fig. 2:**
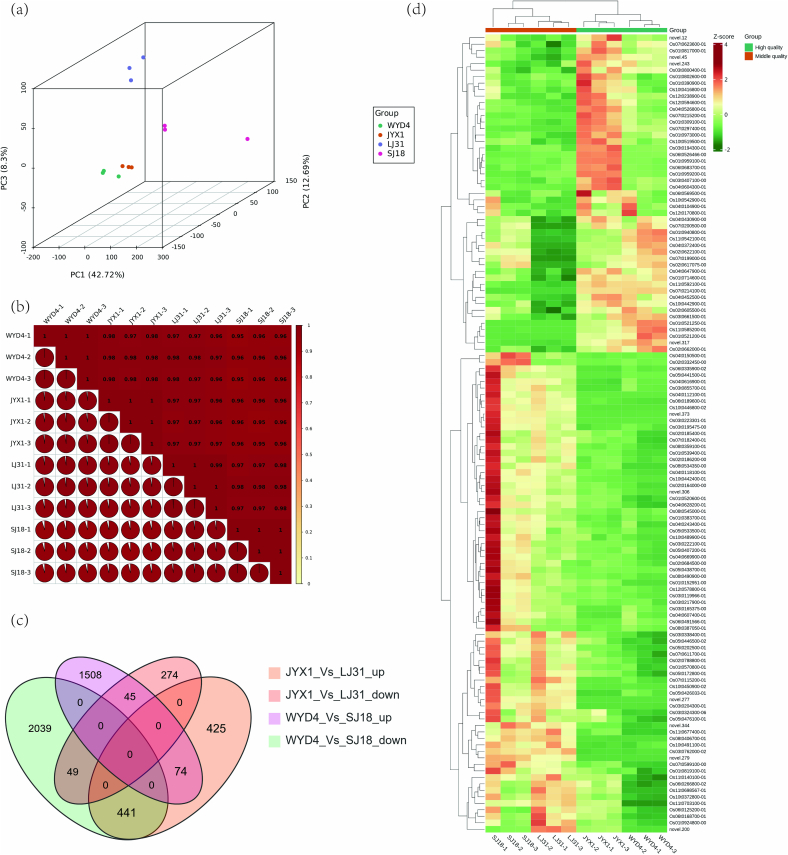
Different expressional genes were identified from WYD4, JYX1, SJ18, and LJ31 varieties. (a) PCA analysis of transcripts identified from the four varieties. Each variety was subjected to three biological replicates. (b) The repeat correlation among the four varieties is evaluated using Pearson’ s correlation coefficient. (c) Venn distribution of different expressional genes identified from JYX1 Vs. LJ31 and WYD4 Vs. SJ18. (d) Heat maps showing different expressional genes from JYX1 Vs. LJ31 and WYD4 Vs. SJ18.

Total RNA from three biological replicates was subjected to RNA-Sequencing (RNA-seq) analysis to elucidate the differences between premium and second-best groups. Over 67 million raw reads were obtained in each sample and more than 65.92 million clean reads were obtained per library, with approximately 94 % uniquely mapped to the rice genome. The quality scores of Q20 and Q30 were > 97 % and 91 %, respectively (Table S4). A total of 32,813 genes from four varieties were detected by RNA-Seq analysis (Table S5). These results demonstrate we obtained high-quality RNA-seq data. As illustrated in Fig. 2, PCA was conducted to ascertain the contributions of PC1, PC2, and PC3, which were found to be 42.72 %, 12.69 %, and 8.3 %, respectively. The combined contribution of the two principal components was 55.41 %. The dots of the same color were from the same sample group, indicating clear distinctions among the four groups (Fig. 2a). According to the transcripts, JYX1 exhibited the closest relationship with WYD4, as did SJ18 and LJ31 (Fig. 2b). Following the screening process, 4156 and 1308 distinct expressional genes were identified in the comparison between WYD4 Vs. SJ18 and WYD4 Vs. LJ31, as identified by *p*-value ≤0.05 and |log2 Fold Change| ≥ 1 (Table S6). Among the identified genes, 123 differentially expressed genes, including 74 up-regulated and 49 down-regulated genes, were found in WYD4 Vs. SJ18 and WYD4 Vs. LJ31 rice groups, respectively (Fig. 2c and d).

**Fig. 3:**
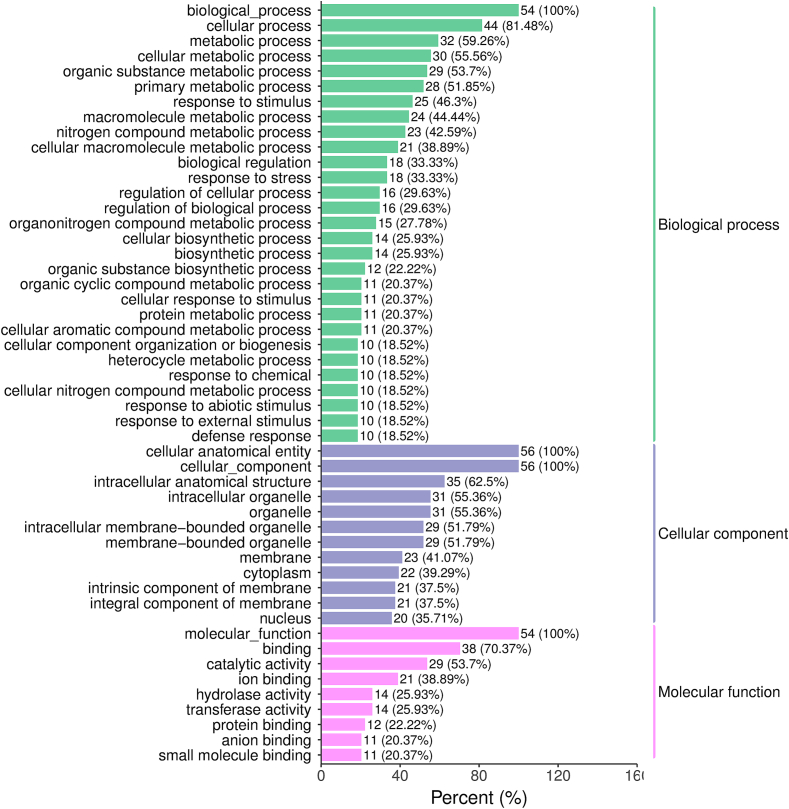
GO enrichment analysis of different expressional genes in the premium rice group.

The GO analysis revealed that the annotations of the various expressional genes were concentrated in a multitude of metabolic and biosynthetic processes, including organic substance metabolic and biosynthesis processes, macromolecule metabolic processes, nitrogen and organonitrogen compound metabolic processes, protein metabolic processes, and organic substance metabolic and biosynthesis processes. The above different expressional genes were membrane-related components that functioned in catalytic activity and binding of protein and small molecule binding (Fig. 3). This result offers a comprehensive overview of the diverse expressional genes involved in rice quality formation. KEGG analysis enriched 34 pathways, including 59 genes, and identified 9 pathways associated with amino acid biosynthesis or metabolism. Other enrichment metabolism pathways were carbon, lipid, and sulfur metabolism, and biosynthesis pathways of secondary metabolites, pantothenate, folate, diterpenoid, ubiquinone, and ribosome-related pathways. In addition, pathways associated with damage and repair, signaling, and circadian rhythm were also enriched (Table 3). This finding suggests that milled rice exhibits metabolic activity and contains essential components.

**Table 1:** The characterization of four rice varieties from Northeast China.

Physicochemical properties	WYD4	JYX1	LJ31	SJ18
Apparent amylose content (%)	17.8	16.46	17.5	14.9
Protein content (%)	5.6	5.6	6.5	6.6
Eating quality (score)	86.7	84.0	79.8	82.5
Seed length (mm)	6.1	4.6	4.8	5.4
Seed width (mm)	2.3	2.7	2.8	2.6
Chalkiness degree (%)	1.8	1.4	0.9	0.2
Head rice rate (%)	91	98.8	96.6	96.4
Peak Viscosity	186.1	187.9	194.3	259.4
Holding Viscosity	111.59	109.17	123.5	135.7
Breakdown	74.5	78.8	70.8	123.8
Final Viscosity	213.1	198.3	222.8	236.2
Setback	27.0	10.3	28.4	−23.3
Peak Time (min)	5.9	5.8	5.93	5.9
Pasting Temperature (°C)	87.6	87.4	88.4	76.8

**Table 2:** Differential metabolites in WYD4-JYX1 compared with SJ18 and LJ31.

Class II	Compounds	Index	Formula	Vs. LJ31			Vs. SJ18		
				VIP	P-value	Log2 Fold Change	VIP	P-value	Log2 Fold Change
Amino acids and derivatives	L-Arginine	MWSmce119	C6H14N4O2	1.62	0.00	1.17	1.32	0.00	1.23
	Tyr-His-Leu	MW0158484	C21H29N5O5	1.59	0.02	1.12	1.20	0.01	1.74
	Lys-Tyr-Gln	MW0153174	C20H31N5O6	1.52	0.06	−1.24	1.32	0.05	−2.94
	L-Aspartic Acid	mws0219	C4H7NO4	1.52	0.03	−1.18	1.35	0.01	−2.76
	Thr-Ile-Asp-Phe-Glu	MW0157677	C28H41N5O11	1.56	0.03	−2.57	1.25	0.07	−2.30
Ditepenoids	Momilactone B	Wadp009007	C20H26O4	1.56	0.01	−1.11	1.33	0.01	−1.94
Phenolic acids	1-O-Caffeoylglycerol	Hmbp003234	C12H14O6	1.19	0.15	−1.99	1.00	0.05	−1.60
Saccharides	DMelezitose O-rhamnoside	pmb2653	C24H42O20	1.56	0.03	−1.32	1.34	0.01	−2.50

**Table 3:** KEGG pathways of the different expressional genes.

ID	KEGG pathway	Transcripts
ko01100	Metabolic pathways	Os04t0628200–01, Os08t0545000–01, Os10t0542900–01, Os04t0669900–00, Os03t0165375–00, Os02t0605500–01, Os10t0446800–02, Os05t0533500–01
ko04141	Protein processing in the endoplasmic reticulum	Os12t0594600–01
ko00380	Tryptophan metabolism	Os04t0118100–01
ko00270	Cysteine and methionine metabolism	Os04t0669900–00, Os05t0533500–01
ko00290	Valine, leucine, and isoleucine biosynthesis	Os10t0446800–02
ko00330	Arginine and proline metabolism	Os04t0118100–01
ko00360	Phenylalanine metabolism	Os04t0118100–01
ko01230	Biosynthesis of amino acids	Os10t0446800–02, Os05t0533500–01
ko01210	2-Oxocarboxylic acid metabolism	Os10t0446800–02
ko00520	Amino sugar and nucleotide sugar metabolism	Os10t0542900–01
ko01200	Carbon metabolism	Os05t0533500–01
ko01110	Biosynthesis of secondary metabolites	Os04t0104900–01, Os02t0186200–00, Os02t0185400–01, Os06t0569500–01, Os04t0628200–01, Os08t0359100–01, Os02t0605500–01
ko00940	Phenylpropanoid biosynthesis	Os04t0628200–01
ko00941	Flavonoid biosynthesis	Os04t0104900–01
ko00561	Glycerolipid metabolism	Os08t0359100–01
ko00564	Glycerophospholipid metabolism	Os08t0359100–01
ko00770	Pantothenate and CoA biosynthesis	Os10t0446800–02
ko00790	Folate biosynthesis	Os08t0545000–01
ko00130	Ubiquinone and other terpenoid-quinone biosynthesis	Os02t0605500–01
ko00904	Diterpenoid biosynthesis	Os02t0186200–00, Os02t0185400–01, Os06t0569500–01
ko00920	Sulfur metabolism	Os05t0533500–01
ko00196	Photosynthesis-antenna proteins	Os03t0165375–00
ko02010	ABC transporters	Os10t0442900–01
ko03008	Ribosome biogenesis in eukaryotes	Os03t0762000–02
ko03010	Ribosome	Os01t0924800–00
ko03030	DNA replication	Os03t0855700–01
ko03420	Nucleotide excision repair	Os03t0855700–01
ko03430	Mismatch repair	Os03t0855700–01
ko04016	MAPK signaling pathway-plant	Os03t0324300–06, Os06t0526466–00, Os10t0542900–01
ko04075	Plant hormone signal transduction	Os07t0182400–01, Os01t0819100–01, Os04t0604300–01, Os03t0324300–06, Os06t0526466–00
ko04120	Ubiquitin mediated proteolysis	Os12t0594600–01
ko04130	SNARE interactions in vesicular transport	Os08t0534350–00
ko04626	Plant-pathogen interaction	Os04t0112100–01, Os01t0520600–01, Os07t0599100–00
ko04712	Circadian rhythm-plant	Os03t0762000–02

### 3.4 Integrated pathway analysis of differential metabolites and different expressional genes

**Fig. 4:**
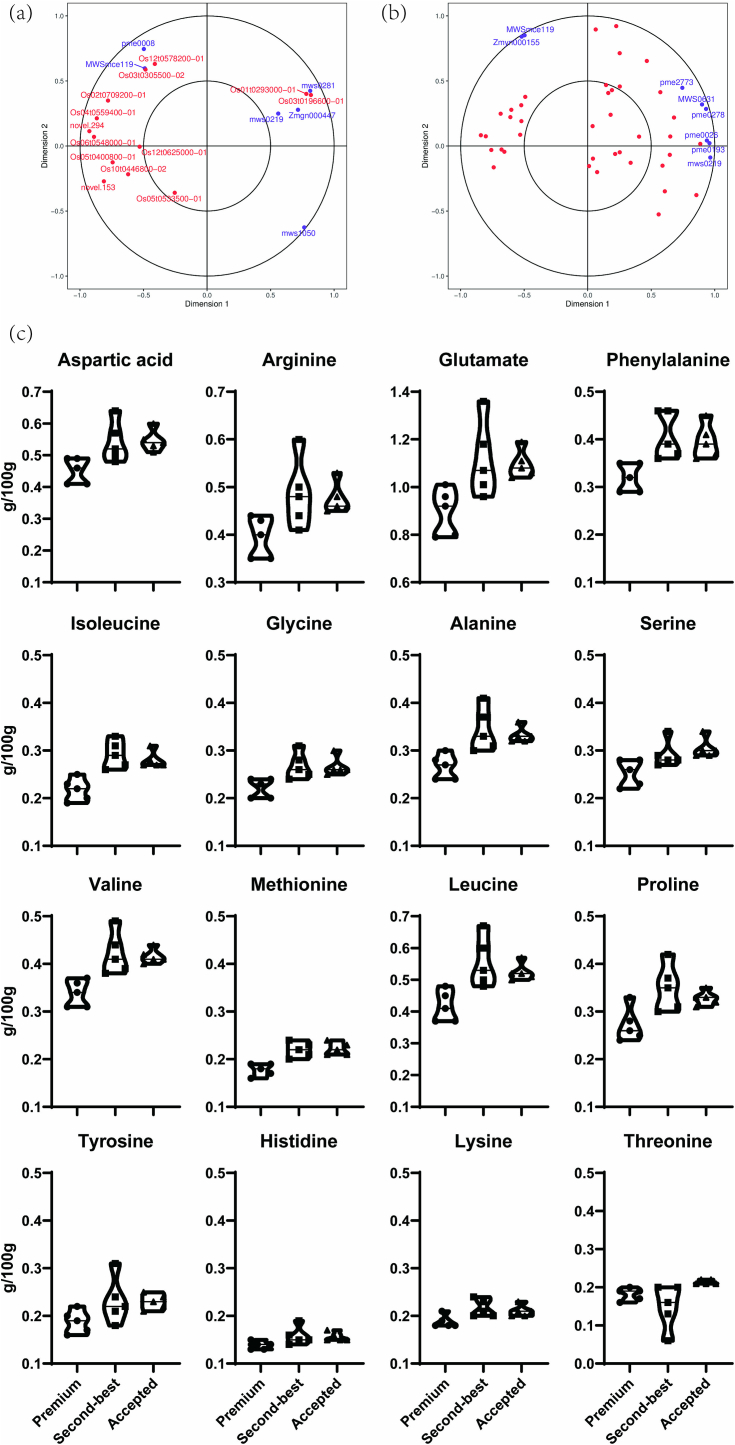
Canonical correlation analysis of metabolites and transcripts from JYX1 Vs. LJ31 and WYD4 Vs. SJ18. (a) Canonical correlation analysis of JYX1 Vs LJ31. The purple dots of metabolites were mws1050 (*O*-acetyl serine), pme0008 (L-citrulline), MWSmce119 (L-arginine), Zmgn000447 (3-Phospho-D-glyceric acid), mws0219 (L-aspartic acid), mws0281 (citric acid). (b) Canonical correlation analysis of WYD4 Vs SJ18. (c) The amino acid content in milled rice varieties was evaluated as premium, second-best, and accepted groups. Each group contains 5 varieties with a similar eating score. Each variety included three replicates. Purple dots represent metabolites, and red pots represent transcripts. Purple dots of metabolites were pme0026 (l-lysine), Zmyn000155 (N-α-acetyl-L-ornithine), pme2773 (L-cystathionine), MWSmce119 (L-arginine), MWS0631 (S-sulfo-L-cysteine), pme0278 (2,6-diaminopimelic acid), pme0193 (l-glutamine), and mws0219 (L-aspartic acid). The red pots were Os01t0232700–01, Os01t0652600–01, Os10t0446800–02, Os05t0429000–01, Os01t0720700–01, Os05t0533500–01, Os03t0126000–01, Os07t0617901–00, Os02t0709200–01, Os01t0725000–01, Os05t0558400–01, Os10t0571200–01, Os12t0145700–01, Os10t0442100–00, Os02t0657600–00, Os01t0723600–01, Os04t0467550–00, Os02t0608550–00, Os03t0826500–01, Os03t0718000–01, Os07t0182100–01, Os04t0254000–01, Os03t0254800–01, Os05t0591500–01, Os06t0564500–01, Os06t0564300–01, novel.153, Os01t0841600–01, Os02t0751800–01, Os01t0764400–01, Os12t0578200–01, Os03t0712800–02, Os12t0145100–01, Os06t0265000–01, Os10t0523700–01, Os06t0548000–01, Os06t0708832–00, Os11t0138600–00, and Os01t0814800–01. (For interpretation of the references to color in this figure legend, the reader is referred to the web version of this article.)

To verify crucial metabolites and transcripts in premium rice, we performed a canonical correlation analysis using metabolomic and transcriptomic data from JYX1 Vs. LJ31 and WYD4 Vs. SJ18. In the same region, the points displayed were far away from the original point. The highest relationship was demonstrated between the two variables, which were closely identified (Fig. 4a and b). Eleven differential metabolites and 42 genes into amino acid pathways were conducted. The biosynthesis and metabolism processes of several amino acids are histidine, tryptophan, phenylalanine, methionine, cysteine, alanine, valine, leucine, isoleucine, arginine, glutamine, lysine, and asparagine. In the group of JYX1 Vs. LJ31, 13 transcripts and 6 metabolites were integrated. Similarly, 39 transcripts and 8 metabolites were integrated from WYD4 Vs. SJ18 group. Six transcripts and 2 metabolites were identified in both groups. The transcripts are acetohydroxy acid isomeroreductase domain-containing protein (Os10t0446800–02), serine acetyltransferase 1 (Os05t0533500–01), histidinol-phosphate aminotransferase (Os02t0709200–01), chorismate mutase (Os12t0578200–01), aspartate aminotransferase (Os06t0548000–01), and hypothetical argininosuccinate lyase (novel.153), and metabolites are L-aspartic acid (pme0219), and L-arginine (MWSmce119) (Fig. 4a and b).

**Fig. 5:**
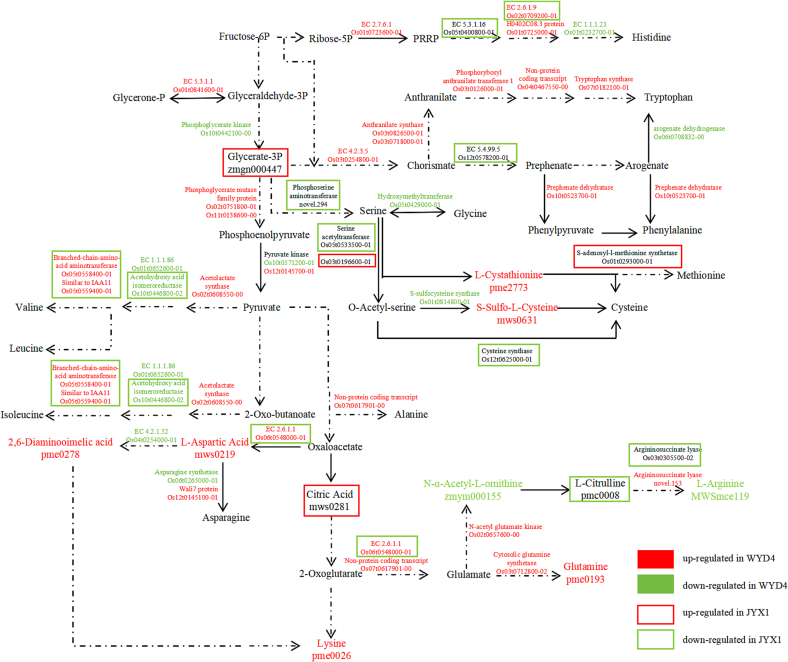
A description of crucial metabolites and genes involved in the amino acid metabolic pathway. Red and green represent up and down-regulation in WYD4. Red and green rectangles represent up and down-regulation in JYX1.

Amino acids are unique taste compounds in cooked rice and serve as the primary substrates for protein synthesis and many metabolic reactions. To verify the effect of the amino acid pathway, we analyzed the content of 16 kinds of hydrolyzed amino acids of rice varieties with different eating quality designated as premium (88.0–89.4), second-best (80.0–80.7) and accepted (71.1–76.2) (Table S7). Except tyrosine, histidine, lysine, and threonine, the content of the other 12 kinds of amino acids was all less accumulated in premium varieties, which made a clear distinction between the premium with the second-best and accepted varieties (Fig. 4). The results demonstrated amino acid is indispensable for regulating the quality of rice, especially aspartic acid, phenylalanine, methionine, alanine, valine, leucine, isoleucine, arginine, glutamate, and aspartic acid metabolic pathway process. The research on crucial metabolites combining the genetic factors of premium rice provided a novel point for quality formation and discrimination detection (Fig. 5).

## 4 Discussion

### 4.1 Wuchang Daohuaxiang rice is well-known for its aroma and palatability taste

Rice is classified into different grades according to its quality. The designation of a product as high-quality or premium is contingent upon its association with a specific human activity (e.g., savoir-faire) and geographic location. In countries where rice is a staple food, specific varieties are considered premium and represent the pinnacle of quality (Champagne et al., 2010). The distinctive texture of premium rice is primarily influenced by the geographical and natural conditions of rice cultivation, the specific rice cultivar, environmental factors, traditional cultivation practices, processing techniques, and storage procedures. In this study, the premium variety, WYD4 is renowned and widely recognized as Wuchang Daohuaxiang 2 (稻花香 in Chinese), which is derived from a sentence in a Song dynasty poem “稻花香里说丰年”, which translates to “The paddy flower aroma heralds a bumper year”. WYD4 has been bestowed the gold medal from 2018 to 2022 in the “Chinese Rice Festival” due to its palatability and aroma. Similarly, the JYX1 variety has been awarded the gold medal on three occasions in the competition of “Evaluation of eating quality of high-quality rice varieties in China”. LJ31 and SJ18, as ordinary and aromatic varieties, exhibited the most significant extension areas in China, respectively. Consequently, the quality of different rice cultivars is highly analogous to that of the same cultivation traditions and processing procedures. Our team identified the quality parameters of these varieties (Table S1). Official signage is crucial in identifying these premium products, particularly, in terms of, quality and origin. The study revealed the existence of rice varieties with high degrees of similarity and delineated the criteria for representativeness and universality standards.

### 4.2 Metabolomics reveals the candidate biomarkers for premium rice

Metabolomic technology employs high-throughput chemometrics to qualitatively and quantitatively assess the presence and abundance of small-molecule metabolites in biological samples, thereby providing data for the analysis and discrimination of critical metabolites in metabolic pathways (Uawisetwathana & Karoonuthaisiri, 2019). In this study, 757 metabolites were typically identified (Table S2), whereas 228 different metabolites were used for further identification (Table S3). However, only eight different metabolites (Fig. 2c) were identified and these fewer targets may be attributed to similar seasons, weather conditions, or geographic environments. However, different cultivars demonstrate dynamic alterations in the quantity and constitution of metabolites and gene expression. Among these metabolites, amino acids, carbohydrates, and lipids play a pivotal role in taste perception and may contribute to the overall sensory quality of the rice (Chen et al., 2023; Kochem, 2017; Xiong et al., 2022). Amino acid metabolites may play a pivotal role in the quality formation of premium rice varieties (Xiong et al., 2023). For instance, the shikimate and pyruvate metabolic pathways in the amino acid pathway have been demonstrated to be associated with the head-milled rice rate (Yang, Shen, et al., 2022). It has been proved that arginine biosynthesis, arginine, and proline metabolism were enriched in high-quality rice varieties (Zhu et al., 2022). The amino acids, L-arginine, Tyr-His-Leu, L-aspartic acid, and Thr-Ile-Asp-Phe-Glu, were different in the premium and second-best groups (Table 2). Most of the amino acids are derived from free and seldom are hydrolytic amino acids. However, the low content of free amino acids in milled rice is adverse to obtaining more accurate data for daily detection. As a result, we selected hydrolytic amino acids as biomarkers to verify, that most of the amino acids were lower in premium rice (Fig. 4c). Because most of the amino acids were mainly hydrolyzed from glutelin, which is negative with eating quality (Yang, Jiang, et al., 2022). This result demonstrated amino acids could be identified as the potential markers (Zhao et al., 2023) for differentiating premium rice in Northeast China.

Additionally, essential carbohydrate metabolites, including sucrose, levan, and amylose, were utilized to produce high-quality rice (Zhu et al., 2022). The research identified carbohydrates, DMelezitose O-rhamnoside (Table 2) distinguishing accumulated premium rice. Moreover, lipids were identified as pivotal metabolites and linked to the development formation of rice quality within the metabolic pathway (Zhu et al., 2022). Our research identified 1-O-caffeoyl glycerol (Table 2) is derived from the lipid pathway. Furthermore, the diterpenoids component, momilactone B (Table 2), which has allelopathic activity and can inhibit the growth of competing plants, was also identified. Nevertheless, the complete momilactone biosynthetic pathway remains elusive (De La Peña & Sattely, 2021). These distinct metabolites may serve as potential biomarkers following the verification process through the chemometric method. The further utilization of metabolomic technology to explore rice quality-related metabolites in other rice varieties is important for breeding new high-quality rice varieties (Li, Yang, et al., 2022).

### 4.3 Combination of metabolomics and transcriptomics analysis implies the amino acid pathway plays a vital role in distinguishing premium and second-best rice

This study aimed at the gene regulatory network and metabolic pathways that control rice quality. As previously reported, the RNA of the milled rice was severely degraded (Yang, Shen, et al., 2022). In our study, milled rice as primarily starch-rich endosperm, was promptly collected from unhusked rice for transcriptomic analysis. It was in a state of dormancy but still alive. As a result, we obtained high-quality data comprising 32, 813 expressed genes (Table S5). In our research, 5464 different expressional genes were identified and compared between the premium and second-best varieties (Fig. 2), indicating that the gene expressions within similar groups exhibited differential patterns. To gain further insight into the transcriptional mechanisms underlying the formation of high-quality rice, the different expressional genes derived from the same group were subjected to 34 KEGG pathway enrichment analyses. This revealed that these genes are involved in a multitude of metabolic and biosynthetic processes, such as damage and repair, signal transduction, and circadian rhythm-related pathways (Table 2). This is because the starchy endosperm cells die upon seed maturation and desiccation. However, the tissue remains alive with the capacity for some metabolic activities, including a redox system crucial for supporting and nurturing the developing embryo (Doll & Ingram, 2022). The different expressional genes investigated in this research were enriched in amino acid metabolism-related pathways, as illustrated in Fig. 3 and Table 3. Amino acids constitute proteins, the second most significant component of rice endosperm, accounting for approximately 10 % of the weight of milled rice. The content and amino acid composition of rice significantly impact its quality (Duan & Sun, 2005). Similarly, different expressional genes and metabolites were integrated into amino acid pathways (Fig. 4 and Fig. 5), indicating that metabolically biased amino acid pathways may be associated with producing high-quality rice. It was anticipated that these compounds and pathways would benefit rice breeders seeking to enhance the quality of their crops. To date, there needs to be more research examining the relationship between metabolites and transcripts and rice quality except for studies on milled rice.

## 5 Conclusion

This study performed a comprehensive metabolomic and transcriptomic analysis to identify the essential chemical compositions and gene expression pathways in four conventional Japonica rice varieties. The results indicated that the formation of premium japonica rice is associated with the amino acid pathway. The composition and concentration of amino acids and derivatives, such as aspartic acid, phenylalanine, methionine, alanine, valine, leucine, isoleucine, arginine, and glutamate could be potential biomarkers for differentiating premium rice in Northeast China. This study yielded new insights into the formation mechanism of high-quality rice and provided primary data for the identification of premium rice identification and metabolomics-assisted breeding.

The following are the supplementary data related to this article.

Table S1 The eating quality, apparent amylose, and protein content of four varieties since the year of cultivar registration to 2022. Table S1

Table S2 757 differential metabolites from four varieties from Northeast China. Table S2

Table S3 Differential metabolites in WYD4 Vs. SJ18 and JYX1 Vs. LJ31 groups Table S3

Table S4 Data quality of reading count of four varieties. Table S4

Table S5 Total expressed transcripts identified by RNA-seq. Table S5

Table S6 Different expressional genes were identified in WYD4 Vs. SJ18 and WYD4 Vs. LJ31 rice groups. Table S6

Table S7 Selected rice varieties were evaluated as premium, second-best, and accepted groups. Table S7

## Funding

This work was supported by the 10.13039/501100005046Natural Science Foundation of Heilongjiang Province [YQ2022C032], Scientific Research Business Expenses of Heilongjiang Provincial Scientific Research Institutes [CZKYF2024–1-C001], Heilongjiang Academy of Agricultural Sciences Postdoctoral Research and Introduction of Talent Research Initiation Fund, and Heilongjiang Agricultural Science and Technology Innovation Leapfrog Project Agricultural Science and Technology Innovation Key Project “Optimization integration and application of PREMIUM standard system”.

## CRediT authorship contribution statement

**Jing Wang:** Writing – review & editing, Conceptualization. **Haitao Guan:** Supervision. **Xiaolei Zhang:** Data curation. **Changjun Dai:** Data curation. **Cuiling Wang:** Investigation. **Guofeng Chen:** Formal analysis, Data curation. **Kun Li:** Formal analysis, Data curation. **Zhenhua Xu:** Investigation, Data curation. **Ruiying Zhang:** Writing – original draft, Methodology. **Baohai Liu:** Writing – original draft, Resources. **Hongtao Wen:** Writing – original draft, Resources.

## Declaration of competing interest

The authors declare the following financial interests/personal relationships which may be considered as potential competing interests: Jing Wang reports statistical analysis was provided by Wuhan Metware Biotechnology Co., Ltd. If there are other authors, they declare that they have no known competing financial interests or personal relationships that could have appeared to influence the work reported in this paper.

## Data Availability

Data will be made available on request.

## References

[bb0005] Barbano D.M., Clark J.L., Dunham C.E., Flemin R.J. (1990). Kjeldahl method for determination of total nitrogen content of milk: Collaborative study. Journal of the Association of Official Analytical Chemists.

[bb0010] Champagne E.T., Bett K.L., Vinyard B.T., McClung A.M., Barton F.E., Moldenhauer K., McKenzie K. (1999). Correlation between cooked rice texture and rapid visco analyser measurements. Cereal Chemistry.

[bb0015] Champagne E.T., Bett-Garber K.L., Fitzgerald M.A., Grimm C.C., Lea J., Ohtsubo K.I., Reinke R. (2010). Important sensory properties differentiating premium rice varieties. Rice.

[bb0020] Charoenthaikij P., Chaovanalikit A., Uan-On T., Waimaleongora-ek P. (2021). Quality of different rice cultivars and factors influencing consumer willingness-to-purchase rice. International Journal of Food Science & Technology.

[bb0025] Chen G.Y., Li G.P., Li C.M., Tu Y.B., Lan Y., Wu C.Y., Li T. (2023). Effects of the potassium application rate on lipid synthesis and eating quality of two rice cultivars. Journal of Integrative Agriculture.

[bb0030] Custodio M.C., Cuevas R.P., Ynion J., Laborte A.G., Velasco M.L., Demont M. (2019). Rice quality: How is it defined by consumers, industry, food scientists, and geneticists?. Trends in Food Science & Technology.

[bb0035] Daygon V.D., Calingacion M., Forster L.C., Voss J.J.D., Fitzgerald M.A. (2017). Metabolomics and genomics combine to unravel the pathway for the presence of fragrance in rice. Scientific Reports.

[bb0040] De La Peña R., Sattely E.S. (2021). Rerouting plant terpene biosynthesis enables momilactone pathway elucidation. Nature Chemical Biology.

[bb0045] De Vos R.C., Moco S., Lommen A., Keurentjes J.J., Bino R.J., Hall R.D. (2007). Untargeted large-scale plant metabolomics using liquid chromatography coupled to mass spectrometry. Nature Protocols.

[bb0050] Doll N.M., Ingram G.C. (2022). Embryo-endosperm interactions. Annual Review of Plant Biology.

[bb0060] Duan M., Sun S.S. (2005). Profiling the expression of genes controlling rice grain quality. Plant Molecular Biology.

[bb0065] Galindo-Prieto B., Eriksson L., Trygg J. (2015). Variable influence on projection (VIP) for OPLS models and its applicability in multivariate time series analysis. Chemometrics and Intelligent Laboratory Systems.

[bb0070] Guo J., Zhou X., Chen D., Chen K., Ye C., Liu J., Liu C. (2024). Effect of fat content on rice taste quality through transcriptome analysis. Genes (Basel).

[bb0080] Jiang J., Song S., Hu C., Jing C., Xu Q., Li X., Dang X. (2024). QTL detection and candidate gene identification for eating and cooking quality traits in rice (*Oryza sativa* L.) via a genome-wide association study. International Journal of Molecular Sciences.

[bb0085] Kochem M. (2017). Type 1 taste receptors in taste and metabolism. Annals of Nutrition and Metabolism.

[bb0090] Li F., Liu Y., Zhang X., Liu L., Yan Y., Ji X., Kong F., Zhao Y., Li J., Peng T., Sun H., Du Y., Zhao Q. (2022). Transcriptome and metabolome analyses reveals the pathway and metabolites of grain quality under phytochrome B in rice (*Oryza sativa* L.). Rice.

[bb0095] Li Y., Yang Z., Yang C., Liu Z., Shen S., Zhan C., Luo J. (2022). The NET locus determines the food taste, cooking and nutrition quality of rice. Science Bulletin.

[bb0100] Liu X., Liu C.Q., Wang Y.X., Ning M.Y., Jing Q., Zhang C.Y. (2022). Current situation and suggestions on the development of high-quality rice branding in China. China Rice.

[bb0105] Liu Y., Li Y., Peng Y., Yang Y., Wang Q. (2020). Detection of fraud in high-quality rice by near-infrared spectroscopy. Journal of Food Science.

[bb0110] Liu Y., Liu J., Liu M., Strappe P., Sun H., Zhou Z. (2020). Comparative non-targeted metabolomic analysis reveals insights into the mechanism of rice yellowing. Food Chemistry.

[bb0115] Liu Z., Jiang S., Jiang L., Li W., Tang Y., He W., Wang M., Xing J., Cui Y., Lin Q., Yu F., Wang L. (2022). Transcription factor OsSGL is a regulator of starch synthesis and grain quality in rice. Journal of Experimental Botany.

[bb0120] Ramalingam A.P., Mohanavel W., Premnath A., Muthurajan R., Prasad P.V.V., Perumal R. (2021). Large-scale non-targeted metabolomics reveals antioxidant, nutraceutical and therapeutic potentials of sorghum. Antioxidants.

[bb0125] Rana N., Kumawat S., Kumar V., Bansal R., Mandlik R., Dhiman P., Sonah H. (2022). Deciphering haplotypic variation and gene expression dynamics associated with nutritional and cooking quality in rice. Cells.

[bb0130] Setyaningsih W., Majchrzak T., Dymerski T., Namieśnik J., Palma M. (2019). Key-marker volatile compounds in aromatic rice (*Oryza sativa*) grains: An HS-SPME extraction method combined with GC×GC-TOFMS. Molecules.

[bb0135] Uawisetwathana U., Karoonuthaisiri N. (2019). Metabolomics for rice quality and traceability: Feasibility and future aspects. Current Opinion in Food Science.

[bb0140] Wang W.Q., Xu D.Y., Sui Y.P., Ding X.H., Song X.J. (2022). A multiomic study uncovers a bZIP23-PER1A-mediated detoxification pathway to enhance seed vigor in rice. Proceedings of the National Academy of Sciences of the United States of America.

[bb0145] Xiao J., Gu C., He S., Zhu D., Huang Y., Zhou Q. (2021). Widely targeted metabolomics analysis reveals new biomarkers and mechanistic insights on chestnut (*Castanea mollissima* Bl.) calcification process. Food Research Internal.

[bb0150] Xiong Q., Sun C., Shi H., Cai S., Xie H., Liu F., Zhu J. (2022). Analysis of related metabolites affecting taste values in rice under different nitrogen fertilizer amounts and planting densities. Foods.

[bb0155] Xiong Q., Sun C., Wang R., Wang X., Zhang Y., Zhu J. (2023). The key metabolites in rice quality formation of conventional japonica varieties. Current Issues in Molecular Biology.

[bb0160] Yang W., Jiang X., Xie Y., Chen L., Zhao J., Liu B., Zhang S., Liu D. (2022). Transcriptome and metabolome analyses reveal new insights into the regulatory mechanism of head milled rice rate. Plants.

[bb0165] Yang X., Pan Y., Xia X., Qing D., Chen W., Nong B., Deng G. (2023). Molecular basis of genetic improvement for key rice quality traits in southern China. Genomics.

[bb0170] Yang X.Y., Liu Z.H., Li G.H., Wang Q.S., Wang S.H., Ding Y.F. (2013). Determination of rice amylopectin chain-length distribution by using high performance size exclusion chromatography. Scientia Agricultura Sinica.

[bb0175] Yang Y., Shen Z., Li Y., Xu C., Xia H., Zhuang H., Sun S., Guo M., Yan C. (2022). Rapid improvement of rice eating and cooking quality through gene editing toward glutelin as target. Journal of Integrative Plant Biology.

[bb0180] Zeng T., Fang B., Huang F., Dai L., Tang Z., Tian J., Cao G., Meng X., Liu Y., Lei B., Lu M., Cai Z. (2021). Mass spectrometry-based metabolomics investigation on two different indica rice grains (*Oryza sativa* L.) under cadmium stress. Food Chemistry.

[bb0185] Zhang C., Zhu L., Shao K., Gu M., Liu Q. (2013). Toward underlying reasons for rice starches having low viscosity and high amylose: Physiochemical and structural characteristics. Journal of the Science of Food and Agriculture.

[bb0190] Zhao J., Liu W., Chen Y., Zhang X., Wang X., Wang F., Qian Y., Qiu J. (2022). Identification of markers for tea authenticity assessment: Non-targeted metabolomics of highly similar oolong tea cultivars (*Camellia sinensis* var. sinensis). Food Control.

[bb0195] Zhao L., Duan X., Liu H., Zhang D., Wang Q., Liu J., Sun H. (2022). A panel of lipid markers for rice discrimination of Wuchang Daohuaxiang in China. Food Research International.

[bb0200] Zhao L., Liu J., Wang J., Duan X., Hui S. (2023). Key secondary metabolite markers for Wuchang Daohuaxiang rice discrimination in China. Food Research International.

[bb0205] Zhao Q., Lin J., Wang C., Yousaf L., Xue Y., Shen Q. (2021). Protein structural properties and proteomic analysis of rice during storage at different temperatures. Food Chemistry.

[bb0210] Zheng Z., Zhang C., Liu K., Liu Q. (2022). Volatile organic compounds, evaluation methods and processing properties for cooked rice flavor. Rice.

[bb0215] Zhu D., Zheng X., Yu J., Chen M., Li M., Shao Y. (2023). Effects of starch molecular structure and physicochemical properties on eating quality of *Indica* rice with similar apparent amylose and protein contents. Foods.

[bb0220] Zhu J., Li A., Sun C., Zhang J., Hu J., Wang S., Zhou N., Xiong Q. (2022). Rice quality-related metabolites and the regulatory roles of key metabolites in metabolic pathways of high-quality semi-glutinous japonica rice varieties. Foods.

[bb0225] Zhu T., Jackson D.S., Wehling R.L., Geera B. (2008). Comparison of amylose setermination methods and the development of a dual wavelength iodine binding technique. Cereal Chemistry.

